# Cophylogenetic analysis suggests cospeciation between the Scorpion *Mycoplasma* Clade symbionts and their hosts

**DOI:** 10.1371/journal.pone.0209588

**Published:** 2019-01-09

**Authors:** Luis M. Bolaños, Mónica Rosenblueth, Amaranta Manrique de Lara, Analí Migueles-Lozano, Citlali Gil-Aguillón, Valeria Mateo-Estrada, Francisco González-Serrano, Carlos E. Santibáñez-López, Tonalli García-Santibáñez, Esperanza Martínez-Romero

**Affiliations:** 1 Laboratorio de Ecología Genómica, Centro de Ciencias Genómicas, Universidad Nacional Autónoma de México, Cuernavaca, Mor, Mexico; 2 Departamento de Medicina Molecular y Bioprocesos, Instituto de Biotecnología, Universidad Nacional Autónoma de México, Cuernavaca, Mor, Mexico; University of Milan, ITALY

## Abstract

Scorpions are predator arachnids of ancient origin and worldwide distribution. Two scorpion species, *Vaejovis smithi* and *Centruroides limpidus*, were found to harbor two different Mollicutes phylotypes: a Scorpion *Mycoplasma* Clade (SMC) and Scorpion Group 1 (SG1). Here we investigated, using a targeted gene sequencing strategy, whether these Mollicutes were present in 23 scorpion morphospecies belonging to the Vaejovidae, Carboctonidae, Euscorpiidae, Diplocentridae, and Buthidae families. Our results revealed that SMC is found in a species-specific association with Vaejovidae and Buthidae, whereas SG1 is uniquely found in Vaejovidae. SMC and SG1 co-occur only in *Vaejovis smithi* where 43% of the individuals host both phylotypes. A phylogenetic analysis of Mollicutes 16S rRNA showed that SMC and SG1 constitute well-delineated phylotypes. Additionally, we found that SMC and scorpion phylogenies are significantly congruent, supporting the observation that a cospeciation process may have occurred. This study highlights the phylogenetic diversity of the scorpion associated Mollicutes through different species revealing a possible cospeciation pattern.

## Introduction

Many animals possess symbiotic bacteria of mutualistic nature [1]. They have different physiological roles in their hosts, including nutrient uptake and synthesis [2,3] and participate in digestion [4], reproduction [5,6], immune system maturation [7], toxin degradation [8,9], toxin production for prey killing [10], and suppression of other symbionts [11]. In addition, bacterial symbionts offer protection against natural enemies such as pathogenic fungi [12,13], viruses [14], predators [15], parasitoids [16–18], and parasitic nematodes [19]. In some insects such as termites, bark beetles and in the carmine cochineals there are nitrogen-fixing bacteria that compensate low nitrogen diets [20–22].

If mutualistic symbiosis is beneficial for both organisms, transmission of bacterial symbionts to further generations emerges as a mechanism to preserve the advantages of the relationship [23]. Vertical transmission in long evolutionary periods can lead to a cospeciation process [24]. Cospeciation is commonly observed between insects and their bacterial endosymbionts, especially for obligate primary endosymbionts harbored in specialized bacteriocyte cells [25,26]. Cospeciation has been described in many insects of the Hemiptera order [27–36]. However, cospeciation in insects is not limited to bacteriocyte endosymbionts; some gut bacteria can be vertically transmitted and cospeciate with their hosts through different post-hatch mechanisms such as egg smearing, coprophagy, or symbiont capsules [37–39].

Scorpions (Arachnida) are ancient animals that have colonized almost all major landscapes on Earth. They have conserved ancestral anatomical features since terrestrial colonization; these are a clear differentiated metasoma (tail) and mesosoma (body), chelate pedipalps, chelicerae, pectins, and a terminal telson [40]. To date, over 2300 scorpion species have been described worldwide [41,42].

Although bacterial symbionts are recognized as major drivers of evolution in arthropods [43], interactions between scorpions and bacteria have only rarely been studied. Presence of *Wolbachia* has been reported in species from the genus *Opistophthalmus* (Scorpionidae, [44]), *Tityus* (Buthidae, [45]), and *Hemiscorpius* (Hemiscorpiidae, [46]). Notably, prevalent arachnid and insect symbionts such as *Cardinium*, *Rickettsia*, *Spiroplasma*, and *Wolbachia* were undetected with canonical primers in a collection of 40 Vaejovidae scorpion species [47].

Gut bacterial phylotypes of the scorpion species *Centruroides limpidus* and *Vaejovis smithi* represent novel lineages belonging to the Mollicutes. Among these novel lineages, Scorpion Group 1 (SG1) is present with high frequency in *V*. *smithi*, but absent in *C*. *limpidus* specimens. SG1 16S rRNA sequence had 79% identity to *Spiroplasma lampyridicola*. Furthermore, two other closely related *Mycoplasma*-like lineages were found in high frequencies in each of these scorpion species; with 16S rRNA gene sequences 89% and 88% identical to *Mycoplasma hyorhinis*. These lineages form a well-delineated clade within the Mollicutes named Scorpion *Mycoplasma* clade (SMC) [48]. The species-specificity, phylogenetic relationship of SMC lineages within their clade and the recent discovery of Mollicutes in the African scorpion *Androctonus australis* [49], that according to our phylogenetic analysis would correspond to the SMC clade, suggest that this clade might have undergone a cospeciation process with their hosts.

In this study, we expanded the scorpion-symbiont survey to 23 morphospecies belonging to seven genera and five families collected in Central and Southern Mexico. DNA was extracted from gut tissue and screened using Polymerase Chain Reaction (PCR) with specific primers targeting the scorpion Mollicutes. Scorpion gene markers were amplified and sequenced to confirm the taxonomic assignments. Additionally, scorpion and SMC phylogenetic reconstructions were tested to determine whether the topologies reveal cospeciation associations. Here we show that the novel Mollicutes lineages are found as symbionts of a broad group of scorpion species from different locations and habitats. Furthermore, SMC and scorpion phylogenies are significantly congruent, suggesting these organisms have probably undergone a cospeciation process.

## Materials and methods

### Sample collection and DNA extraction

**Fig 1:**
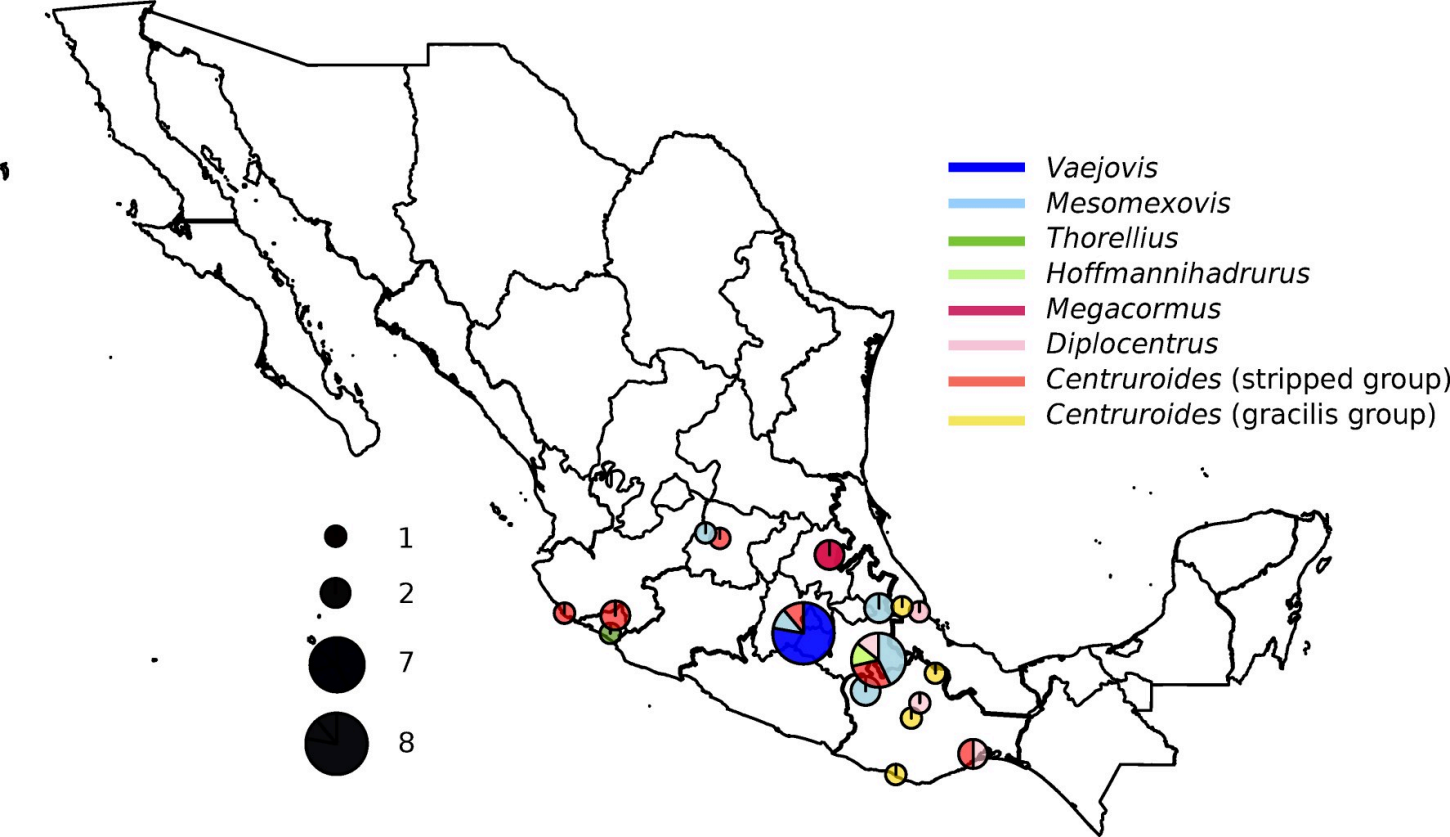
Sampling sites of scorpions in Mexico. Map of Mexico showing the sampling locations, number of individuals and percentage of the different genera collected. Pie chart centres are positioned over the sampling locations. The size of the pie charts corresponds to the number of total individuals collected and colors represent the proportions of the different genera sampled.

Thirty-nine scorpions were collected from different locations in Mexico (Fig 1 and Table 1) during August and September 2015. Sampling conducted in Cuernavaca locality was done in private land under owner’s permission. The rest of the collection was donated from several parties (see Acknowledgments), specifically from other ongoing scorpion projects conducted in Mexico. These specimens have been collected under permits issued by SEMARNAT (SGPA/DGVS/02483 of March 18, 2005) and Scientific Permit FAUT-0175 to Oscar Francke (extended to Carlos Santibáñez from 2014 to 2016). None of the scorpion species analysed in this study are listed as endangered or protected in the Convention on Trade in Endangered Species of Wild Fauna and Flora (CITES). Ethics approval is not required for arachnid-related experimentation. Rock and log rolling was the method to spot the scorpions, thereafter were captured using tweezers and placed in closed containers. The specimens belonged to 23 morphospecies from seven genera and five families. Species identification was based on key morphological characters reported in several scorpion taxonomy studies, using a light stereo microscope; higher genus classification followed Sharma et al. [50]. When not clearly assigned to a species, scorpions were identified as “similar to” (affinis or aff.) the closest morphologically similar species. The mitochondrial 16S rRNA and cytochrome c oxidase subunit 1 (CO1) and the nuclear 28S rRNA scorpion gene sequence analysis confirmed most of the assignments. Due to the diversity and lack of information regarding the starvation stress tolerance for each species, scorpions were processed directly from the field.

**Table 1:** GenBank accession numbers of the DNA sequences obtained by PCR with specific primers for SMC and SG1 (16S rRNA), scorpion mitochondrial gene markers (16S rRNA and CO1) and scorpion nuclear gene marker 28S rRNA.

ID	Species	Sex	Collection site	SMC 16S rRNA	SG1 16S rRNA	Scorpion 16S rRNA	Scorpion CO1	Scorpion 28S rRNA
A9	*Vaejovis smithi*	M	Cuernavaca, Morelos	X	MF134724	MF134669	MF134771	MF134737
A11	*Vaejovis smithi*	ND	Cuernavaca, Morelos	X	MF134725	MF134673	MF134772	MF134738
A3	*Vaejovis smithi*	F	Cuernavaca, Morelos	X	MF134726	MF134670	MF134773	MF134734
A5	*Vaejovis smithi*	F	Cuernavaca, Morelos	X	MF134727	**–**	**–**	**–**
A6	*Vaejovis smithi*	F	Cuernavaca, Morelos	MF134708	MF134728	MF134674	MF134774	MF134735
A7	*Vaejovis smithi*	F	Cuernavaca, Morelos	MF134709	MF134729	MF134671	MF134775	MF134736
A19	*Vaejovis smithi*	ND	Cuernavaca, Morelos	MF134710	MF134730	MF134672	MF134776	X
A32	*Vaejovis granulatus*	M	Cuernavaca, Morelos	X	MF134731	MF134675	MF134777	MF134739
A4	*Mesomexovis* aff. *punctatus*	F	Tepeyehualco, Puebla	MF134712	X	MF134678	MF134782	MF134740
A23	*Mesomexovis* aff. *punctatus*	M	Tepeyehualco, Puebla	MF134711	X	MF134676	MF134784	MF134743
A20	*Mesomexovis* aff. *punctatus*	F	León, Guanajuato	X	MF134722*	MF134681	MF134786	MF134744
A34	*Mesomexovis* aff. *punctatus*	F	Cuernavaca, Morelos	X	MF134723	MF134682	MF134785	MF134745
A17	*Mesomexovis* aff. *subcristatus*	F	Zapotitlán, Puebla	X	X	MF134679	MF134779	MF134746
A26	*Mesomexovis* aff. *subcristatus*	ND	Zapotitlán, Puebla	X	X	MF134677	MF134780	MF134747
A36	*Mesomexovis* aff. *subcristatus*	ND	Zapotitlán, Puebla	X	**–**	MF134680	MF134781	MF134748
A38	*Mesomexovis* aff. *oaxaca*	F	Huajuapam, Oaxaca	X	MF134732	MF134683	MF134783	MF134741
A39	*Mesomexovis* aff. *oaxaca*	F	Huajuapam, Oaxaca	X	**–**	MF134684	X	MF134742
A18	*Thorellius intrepidus*	F	Tecomán, Colima	X	X	MF134685	MF134778	MF134756
A43	*Hoffmannihadrurus aztecus*	F	Zapotitlán, Puebla	X	X	MF134686	MF134787	MF134749
A42	*Megacormus gertschi*	F	Zacualtipan, Hidalgo	X	X	MF134687	MF134805	MF134750
A46	*Megacormus gertschi*	M	Zacualtipan, Hidalgo	X	X	MF134688	MF134806	MF134751
A44	*Diplocentrus duende*	F	Zapotitlán, Puebla	X	MF134733 **	MF134706	X	MF134755
A40	*Diplocentrus tehuano*	F	Santo Domingo Tehuantepec, Oaxaca	X	X	MF134705	MF134788	MF134752
A13	*Diplocentrus mexicanus*	M	San Juan Atepec, Oaxaca	X	X	MF134704	MF134789	MF134753
A22	*Diplocentrus melici*	ND	Idolos, Veracruz	X	X	MF134703	MF134790	MF134754
A16	*Centruroides baergi*	F	Zapotitlán, Puebla	MF134713	X	MF134701	MF134793	MF134764
A47	*Centruroides baergi*	F	Zapotitlán, Puebla	X	**–**	MF134702	MF134794	X
A2	*Centruroides limpidus*	M	Cuernavaca, Morelos	KM978315	X	MF134690	MF134791	MF134760
A12	*Centruroides elegans*	M	Emiliano Zapata, Jalisco	MF134714	X	MF134691	MF134792	MF134762
A14	*Centruroides hoffmanni*	F	Tehuantepec, Oaxaca	X	X	MF134692	MF134795	MF134761
A24	*Centruroides noxius*	M	Pantanal, Nayarit	MF134715	X	MF134698	MF134796	MF134767
A28	*Centruroides noxius*	F	Pantanal, Nayarit	MF134716*	**–**	MF134699	MF134797	MF134768
A15	*Centruroides infamatus*	F	Guanajuato, Guanajuato	X	X	MF134694	MF134798	MF134763
A29	*Centruroides tecomanus*	M	Comalá, Colima	MF134717	**–**	MF134696	MF134800	MF134758
A25	*Centruroides tecomanus*	M	Comalá, Colima	MF134718	X	MF134695	MF134799	MF134757
A30	*Centruroides flavopictus*	F	Xalapa, Veracruz	MF134719	X	MF134700	MF134804	MF134769
A37	*Centruroides fulvipes*	M	Puerto Escondido, Oaxaca	X	X	MF134693	MF134803	MF134765
A10	*Centruroides gracilis*	M	Tuxtepec, Oaxaca	MF134720	X	MF134689	MF134801	MF134759
A41	*Centruroides nigrimanus*	M	Oaxaca, Oaxaca	MF134721	X	MF134697	MF134802	MF134766
Avi	*Mastigoproctus* sp.	ND	Cuernavaca, Morelos	X	MF774367 **	MF134707	MF134807	MF134770

**M**, male; **F**, female; **ND**, not determined; **X**, unamplified sequence; **–** PCR was not performed

* identical sequences obtained from scorpion gut and embryos

** these sequences do not correspond to SG1, but to other spiroplasmas (S1 Fig).

Scorpions were anesthetized by placing them in sealed containers with chloroform and their sex was determined. The exoskeleton surface was disinfected with three rinse cycles of 70% ethanol. Midgut and hindgut (including Malpighian tubules) dissections were performed using a stereoscope, sterile tweezers and scalpel. Occasionally, when pregnant females were dissected, embryos were collected and washed five times with sterile water. DNA was extracted from a pool of embryos from each female. One leg and hemolymph from each specimen were also used for DNA extraction. One leg and the gut of a vinegaroon *Mastigoproctus* sp. were dissected as well.

Dissected tissues were placed in 180 μl of buffer ATL (Qiagen, Valencia, CA). The tissue was macerated with a sterile polypropylene micro pestle to achieve a homogenous solution. DNA extractions were performed using the DNeasy Blood & Tissue kit (Qiagen, Valencia, CA) according to the manufacturer’s recommendations. DNA quality check was performed on 1% agarose gels (90 V, 35 min) and measured with a Nanodrop spectrophotometer (Thermo-Fischer Scientific, Wilmington, DE).

### SMC and SG1 primer design

Primers were designed using OLIGO 7 primer analysis software [51], based on the alignment of SMC and SG1 with *Mycoplasma* and *Spiroplasma* 16S rRNA sequences. The alignment included: 47 representative mycoplasmas and spiroplasmas, 13 sequences obtained from two clone libraries representing SMC from *V*. *smithi* and *C*. *limpidus* (GenBank accession numbers MG813912-MG813923), and the 31 representative SMC and SG1 sequences reported in Bolaños et al. [48]. The alignment corresponds to positions ~37 through 1450 in the *E*. *coli* 16S rRNA sequence. Three primer pairs were chosen: one pair for SMC and two for SG1. The Myco65F and Myco1429R primers have two degenerated positions each, representing variable positions in SMC and mycoplasma sequences; this allowed a certain level of flexibility between different scorpion species. Given that SG1 had only one representative species, we designed two primer pairs to increase the probability of detecting it.

Two sets of primers targeting the *rpoB* gene of SMC and SG1 (S1 Table) were designed with OLIGO 7 based on annotated sequences from *V*. *smithi* and *C*. *limpidus* metagenomes [48] and Mollicutes sequences retrieved from NCBI GenBank

### Polymerase Chain Reaction (PCR) amplifications

DNA extracted from guts, embryos, and hemolymph was used as template for PCR amplification of bacterial 16S rRNA genes. Symbiont targeted PCR amplifications were done with the set of custom primers (Table 2). Mitochondrial (16S rRNA and CO1) and nuclear (28S rRNA) scorpion gene markers were amplified using DNA extracted from one leg as template (Table 2).

**Table 2:** Primers used for PCR amplification.

Primer name (alias)	Sequence (5’ – 3’)	Gene	PCR size (pb)	Annealing temperature (°C)	Extension time	Reference
16Sar (LR-N-13398)	CGCCTGTTTATCAAAAACAT	Invertebrate 16S rRNA	490	47	1:00	Simon et al., 1994 [52]
16Sbr (LR-J-12887)	CTCCGGTTTGAACTCAGATCA	Invertebrate 16S rRNA	490	47	1:00	Simon et al., 1994 [52]
HCO (HCO2198-N-2175)	TAAACTTCAGGGTGACCAAAAAATCA	Invertebrate cytochrome oxidase I	700	43	1:00	Folmer et al., 1994 [53]
LCO (LCO-1490-J-1514)	GGTCAACAAATCATAAAGATATTGG	Invertebrate cytochrome oxidase I	700	43	1:00	Folmer et al., 1994 [53]
28Sa (D3A)	GACCCGTCTTGAAACACGGA	Invertebrate 28S rRNA	330	50	1:00	Nunn et al., 1996 [54]
28Sb (D3B)	TCGGAAGGAACCAGCTACTA	Invertebrate 28S rRNA	330	50	1:00	Nunn et al., 1996 [54]
Myco65F	CRAAYGGGTGAGTAACACGTA	SMC 16S rRNA	1397	54	1:45	This study
Myco1429R	ASGGYTACCTTGTTACGACTT	SMC 16S rRNA	1397	54	1:45	This study
SG1-46F	ACATGCAAGTTGAACGGGAAG	SG1 16S rRNA	1305	54	1:45	This study
SG1-1406R	ATTCACCGCAACGTGGCTGAT	SG1 16S rRNA	1305	54	1:45	This study
SG1F	ACCTAACCTGCCTATATATC	SG1 16S rRNA	1069	54	1:45	This study
SG1R	TTTGTCATCATCCTTTCCTC	SG1 16S rRNA	1069	54	1:45	This study

Final concentrations for 20 μl PCR reactions were as follows: 1 μl DNA (25 ng μl^−1^), 0.2 nM of each primer, 0.2 mM dNTPs, 0.5 U of Taq DNA Polymerase (Invitrogen, Carlsbad, CA, USA), 1X Taq polymerase buffer and 1.5 mM MgCl. The reaction conditions were 94°C for 5 minutes, 30 cycles at 94°C for 1 minute, annealing for 1 minute (see Table 2 for temperatures specific to each primer set), and a final extension at 72°C for 10 minutes. PCR products were observed on 1% agarose gels (90 V, 40 min). Sanger sequencing of correct size amplicons was performed by Macrogen Inc. (Seoul, South Korea). The nucleotide sequences determined in this study have been deposited in GenBank database with accession numbers shown in Table 1, except for *rpoB* sequences which are found in supplementary material (S1 Fasta file).

### Mollicutes phylogenetic analyses

We created a Mollicutes phylogenetic tree with the novel scorpion gut bacteria sequences generated from this study aligned with a set of 16S rRNA gene sequences retrieved from the Ribosomal Database Project (RDP) release 11 [55] and the Silva Database release 128 [56]. The retrieved RDP sequences were type strains, environmental sequences and new sequenced Mollicutes that do not cluster with any of the defined groups, each longer than 1200 bp. Additionally, we used the sequences with GenBank accession numbers KT923413 and KT923388 from the NCBI database. These two sequences represented a *Spiroplasma*-related bacteria and a *Mycoplasma* from *Androctonus australis*, respectively [49]. Sequences were aligned using Mafft 7.397 [57] considering secondary structure. JModelTest 2.1.10 [58] was used to determine the best model to fit the data set. The final alignment consisted of 237 sequences. The Mollicutes phylogenetic tree was constructed on RAXML 8.2.4 [59] using maximum likelihood and the GTR + G model of evolution with 1000 bootstrap replicates; 1069 bases were used for the analysis. The Mollicutes phylogenetic tree was visualized and edited with MEGA7 [60].

A Mollicutes phylogenetic tree based on *rpoB* sequences was constructed with a dataset of 161 sequences retrieved from GenBank. Translation from nucleotide to amino acid sequences was done using the genetic code 4 for *Mycoplasmataceae*. The phylogenetic tree was constructed in PhyML using LG + G + I as model of evolution with 1000 bootstrap replicates; 282 amino acids were used for the analysis. The tree was visualized and edited with MEGA7.

### Scorpion phylogenetic analysis

A scorpion phylogenetic tree was constructed with the three concatenated sequences (16S rRNA, CO1 and 28S rRNA). *A*. *australis* sequences retrieved from GenBank were also included. Sequences were aligned with Clustal W [61]. Only one representative sequence from each scorpion species was included. The tree was constructed and edited with MEGA7 [60], using maximum likelihood and the GTR + G + I model of evolution with 1000 bootstrap replicates; 1209 bases were used for the analysis. Phylogenetic trees with ML were also constructed with each individual gene, using the models suggested by JModelTest 2.1.10. These were T92 + G, GTR + G + I, and HKY for 16S rRNA, CO1 and 28S rRNA, respectively. The tree constructed with the three concatenated genes was selected, given that the phylogeny had a similar topology to both 16S rRNA and CO1. The similarity to the 28S rRNA phylogeny was not considered because this gene by itself does not reflect the phylogenetic relationships of the species, given its high evolutionary conservation.

The Bayesian Inference analyses of the three matrices were performed using MrBayes 3.2.2 [62] and the GTR + G + I model for each partition (16S rRNA, 28S rRNA, and CO1). Four runs each with four Markov chains were implemented for 1 X 10^7^ generations (10 million), using default priors and discarding 2.5 x 10^6^ generations (25%) as burn-in.

### Cophylogenetic analyses

To study the evolutionary associations between SMC and scorpions, we used the maximum likelihood tree topologies obtained from Mollicutes 16S rRNA gene and the three scorpion genes. We used the reconciliation tool Jane 4 [63], which requires costs for five events that can describe host-parasite (or host-symbiont) cophylogenies: cospeciation (joint speciation with the host lineage); duplication (both symbionts are kept in the same host); duplication and host switch (symbionts are duplicated and transferred from one host species to another); losses (loss of symbiont); and failure to diverge. We used the following cost ranges: cospeciation, 0-1; duplication, 1-2; duplication and host switch, 1-2; losses, 1-2; and failure to diverge, 1-2. We used different host ranges in order that Jane computed solutions for every combination of costs. Given that Jane 4 does not consider bootstrap values, nodes lacking substantial support were collapsed, with a cut-off bootstrap value of 50%, so that only nodes with bootstraps greater than 50% were considered for the analysis. Collapsed phylogenies that had been obtained with the different models recreated the same topology. To estimate whether the reconstruction results are different from those expected by chance a thousand random cycles were performed. It must be noted that in the case of *C*. *limpidus*, the scorpion sequences and the SMC sequence were obtained from different specimens, although both were collected at the same location (Cuernavaca, Morelos).

### Genetic and geographic distances similarity analysis

The relationship between geographical localization and positive SMC amplicon was tested using two distance matrices. The first distance matrix was created with the linear distances in kilometers between the different sampling locations. The second was a presence/absence matrix, in which we assigned a value of 0.01 when two samples amplified SMC and 0.99 when just one or none of them were positive for SMC, reflecting the shortest and longest possible distances.

The relationship between geographical localization and genetic distance of scorpions or SMC was tested using the subset of geographical distances in kilometers for SMC positive scorpions. The Kimura 2-parameter genetic distance matrix for scorpions was done with the dnadist program of Phylip package version 3.695 [64]. Distances were calculated using the concatenated alignment of CO1 and 16S rRNA genes. 28S rRNA was excluded from the concatenation due to the absence in one sample. The SMC genetic distance matrix was obtained using the aligned 16S rRNA sequences following the same strategy described for the scorpion genes.

Euclidean distance matrices were calculated with the dist function in R for every matrix. Mantel tests were performed with the mantel command of Vegan package version 2.4-5 [65] for the following pairwise comparisons: geographical distances - SMC presence/absence; geographical distances - scorpion genetic distances; geographical distances - SMC genetic distances; and scorpion - SMC genetic distances.

## Results

### Scorpion *Mycoplasma* clade (SMC) bacteria frequently present in Vaejovidae and Buthidae species, SG1 constrained to Vaejovidae

Thirty-nine scorpions belonging to five families, seven genera and 23 morphospecies were sampled and evaluated by PCR for the presence of the recently discovered Mollicutes SMC and SG1 (Table 1), using specific primers. Mollicutes were found in the guts of eight *Centruroides*, four *Mesomexovis*, two *Vaejovis*, and one *Diplocentrus* species. SMC amplicons were obtained from *Vaejovis smithi* and *Centruroides limpidus* as previously reported [48]. Moreover, SG1 was also found in *Mesomexovis* aff. *punctatus* and eight *Centruroides* species. SG1 was found in *V*. *smithi* as previously reported [48], and in the species *M*. aff. *punctatus* and *Vaejovis granulatus*. SG1 could not be detected in any *Centruroides* spp.

In 43% of *V*. *smithi* scorpions, both SMC and SG1 were found in the same specimen. These frequencies are similar to those previously reported with universal 16S rRNA primers [48]. This was not observed in the *Mesomexovis* species sampled, as they had either SMC or SG1. SMC was detected in two specimens of *M*. aff. *punctatus* (A4 and A23), and SG1 in other two (A20 and A34). SMC and SG1 were not detected in *M*. aff. *subcristatus*, and SG1 was detected in *M*. aff. *oaxaca*.

SMC and SG1 were not detected in *Diplocentrus* spp. We could only amplify a PCR product from one species, *Diplocentrus duende* (A44) with the SG1 primers, but this sequence corresponds to a canonical spiroplasma from the Citri-Chrysopicola-Mirum clade rather than to SG1 (Table 1 and S1 Fig). No PCR amplification products were obtained from *Thorellius intrepidus*, *Hoffmannihadrurus aztecus*, and *Megacormus gertschi* using either the SMC or SG1 primers. There is no clear pattern suggesting a sex-bias in the presence of these bacteria in scorpions (Table 1). DNA extracted from scorpion’s hemolymph did not amplify any of the two Mollicutes symbionts. Overall, 16S rRNA primers used in this study had low frequency of false positives. Only five non–targeted sequences were obtained from the whole set of reactions (S2 Table).

SMC *rpoB* sequences were amplified from five samples from two species, in three *V*. *smithi* specimens and two *Mesomexovis* aff. *punctatus*. Additionally, SG1 *rpoB* sequences were obtained from three *V*. *smithi* specimens and two *Mesomexovis* aff. *punctatus* as well. Amplifications were positive in samples where previously 16S rRNA amplicons of the targeted bacteria were obtained, confirming the presence of the symbionts.

### 16S rRNA phylogeny supported the designation of the novel Scorpion *Mycoplasma* Clade (SMC) and SG1 clade

**Fig 2:**
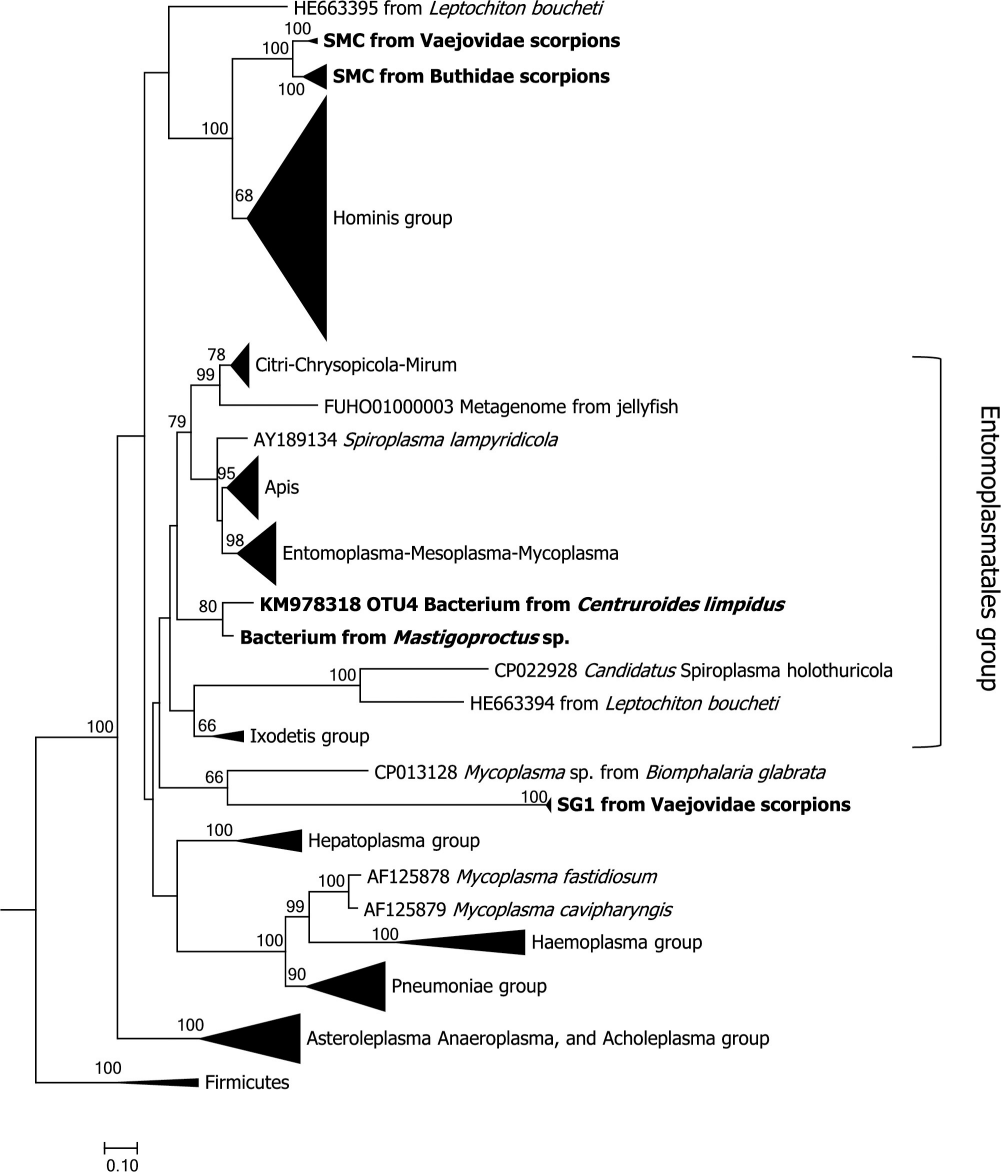
Mollicutes 16S rRNA phylogenetic tree. A phylogenetic tree was constructed with maximum likelihood and the GTR + G model using 237 sequences representing different species and the new lineages found in scorpions. *Streptococcus pneumoniae*, *Bacillus subtilis* and *Bacillus cereus* were used as outgroups. Bootstrap values above 60 are shown. Representative Mollicutes clades were collapsed (See S1 Fig for the non-collapsed tree).

A 16S rRNA phylogenetic tree was constructed using 237 sequences from Mollicutes consisting of the major taxonomic clades including novel SMC, SG1 and scorpion-spiroplasma related sequences (Fig 2 and S1 Fig). SG1 and one of the symbionts from the freshwater snail *Biomphalaria glabrata* seem to form a clade basal to the Entomoplasmatales group. Previously, it was reported that *C*. *limpidus* also harbors a *Spiroplasma* related sequence named OTU4 (KM978318, [48]). This sequence was found in around 30% of *C*. *limpidus* individuals and is 88% similar to *Spiroplasma platyhelix*. It is positioned as a clade within the Entomoplasmatales group, along with a sequence amplified from a vinegaroon (*Mastigoproctus* sp.). Some of the nodes related to these sequences are not well supported (bootstrap values <40), reflecting the divergence between them and the rest of the dataset. It has been challenging to determine the position of SG1 lineage due to the lack of related sequences.

SMC is related to the Mycoplasma Hominis group and sequences within this clade form two major sister subclades. The first one is composed of sequences amplified from scorpions belonging to the Vaejovidae family (genera *Mesomexovis* and *Vaejovis*). The second clade is composed of sequences obtained from scorpions belonging to the Buthidae family (genus *Centruroides* and *Androctonus*). SMC sequences derived from the genus *Centruroides* form a compact clade, and the sequence derived from *A*. *australis* is positioned as a basal group.

Unrelated to the SG1 lineage, the sequence obtained from *Diplocentrus duende* (A44) has a 99% identity with *Spiroplasma leucomae*. Also, *A*. *australis* has a sequence related to the genus *Spiroplasma* (GenBank KT923385). Both sequences are grouped within the canonical Citri-Chrysopicola-Mirum clade and are 99% identical to each other (S1 Fig).

A *rpoB* phylogenetic tree constructed using sequences amplified from *V*. *smithi* and *Mesomexovis* aff. *punctatus* showed SMC embedded within the Hominis group, not as a sister clade of it. SG1 *rpoB* was placed as a sister lineage of the Pulmonis group (S2 Fig).

### Congruence analysis of SMC 16S rRNA and scorpion phylogenies

**Fig 3:**
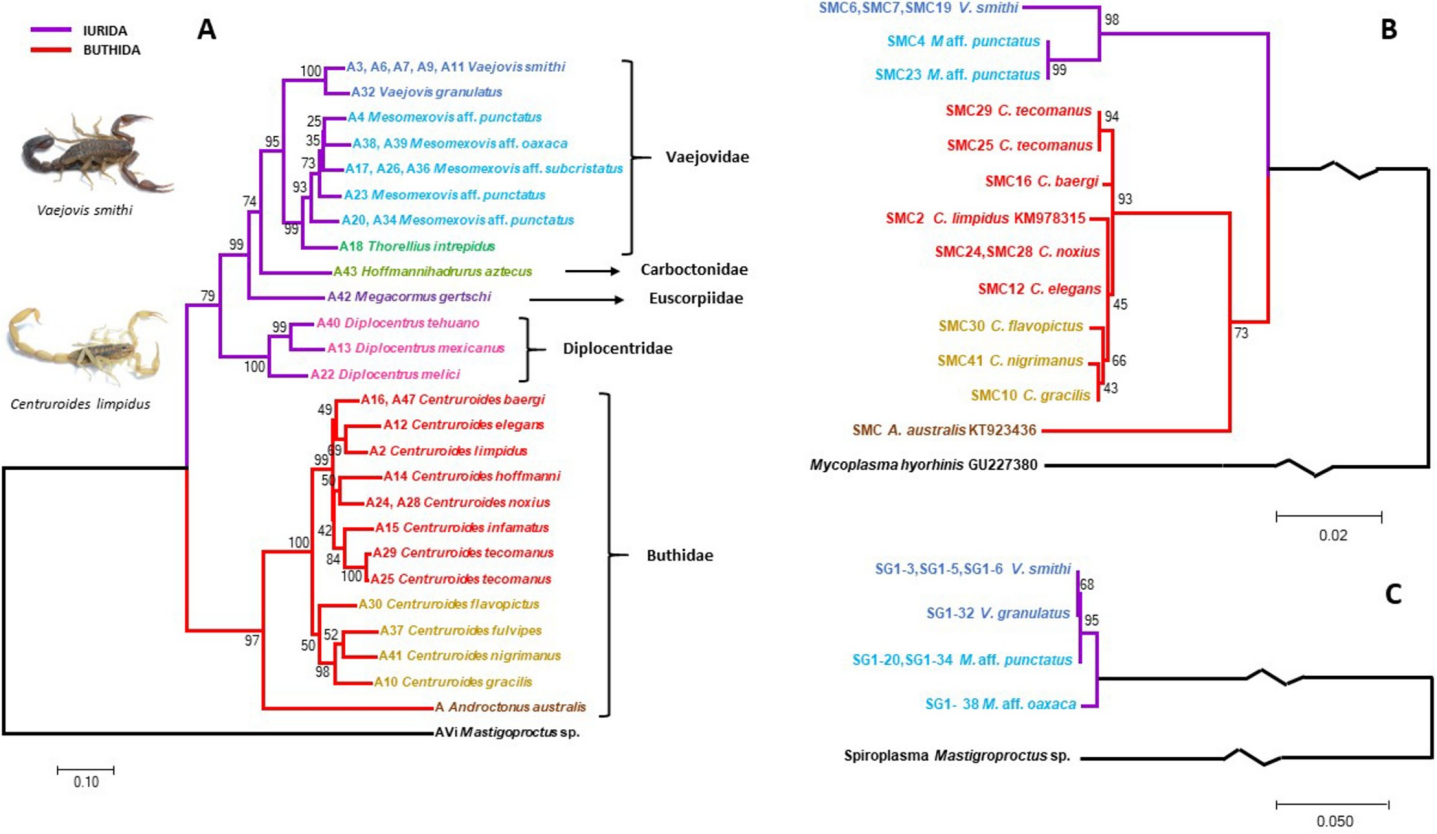
Comparison of scorpion and symbiont phylogenies. Different colors were used for each genus or clade: dark blue, *Vaejovis*; light blue, *Mesomexovis*; dark green, *Thorellius*; light green, *Hoffmanihadrurus*; purple, *Megacormus*; pink, *Diplocentrus*; red, *Centruroides*, “striped group”; yellow, *Centruroides*, *“gracilis* group”; brown, *Androctonus*; black, outgroups (*Mastigoproctus* sp. or *Mycoplasma hyorhinis)*. (A) Scorpion phylogeny with concatenated 16S rRNA, CO1 and 28S rRNA genes (GTR + G + I). (B) SMC 16S rRNA gene phylogeny (GTR + G). (C) SG1 16S rRNA gene phylogeny (GTR + G).

The scorpion phylogenetic trees recovered from the analysis of 16S rRNA, CO1, and 28S rRNA gene sequences based on Bayesian Inference and Maximum likelihood showed congruence with previously published phylogenies (e.g. [49]; Fig 3A, S2 and S3 Figs). For instance, the presence of both currently recognized parvorders (Iurida and Buthida) was recovered in our phylogeny. Moreover, scorpions of the three families Vaejovidae, Carboctonidae and Euscorpiidae form a clade and configure superfamily Chactoidea. Within Vaejovidae, the specimens classified as *Mesomexovis* aff *punctatus*, branched independently in different positions suggesting being three species instead of a monophyletic group representing one species. Diplocentridae is representative of the superfamily Scorpionoidea. These two superfamilies are representatives of parvorder Iurida. Family Buthidae was monophyletic and is representative of parvorder Buthida. Remarkably, the distinct species of genus *Centruroides* used in this study, were recovered in two clades, in agreement with their morphological characterization (“striped group” and “*gracilis* group” *sensu* [66]).

**Fig 4:**
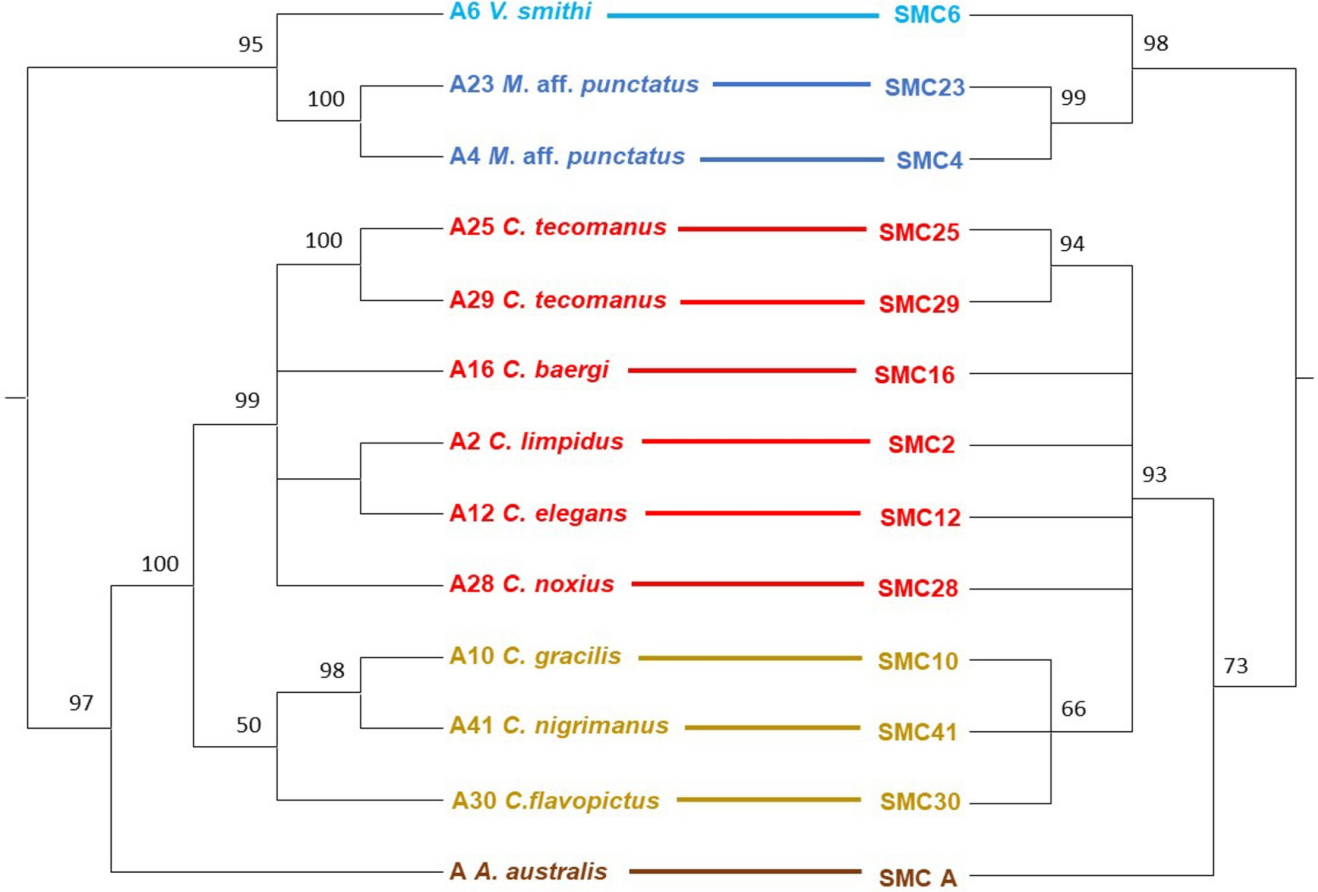
Tanglegrams of host species and SMC phylogenies used for cophylogenetic analyses. Scorpion phylogeny (left) constructed with concatenated 16S rRNA, CO1 and 28S rRNA genes, and SMC phylogeny (right) with 16S rRNA. Nodes with bootstrap values lower than 50% were collapsed. Colors are as in Fig 3.

**Fig 5:**
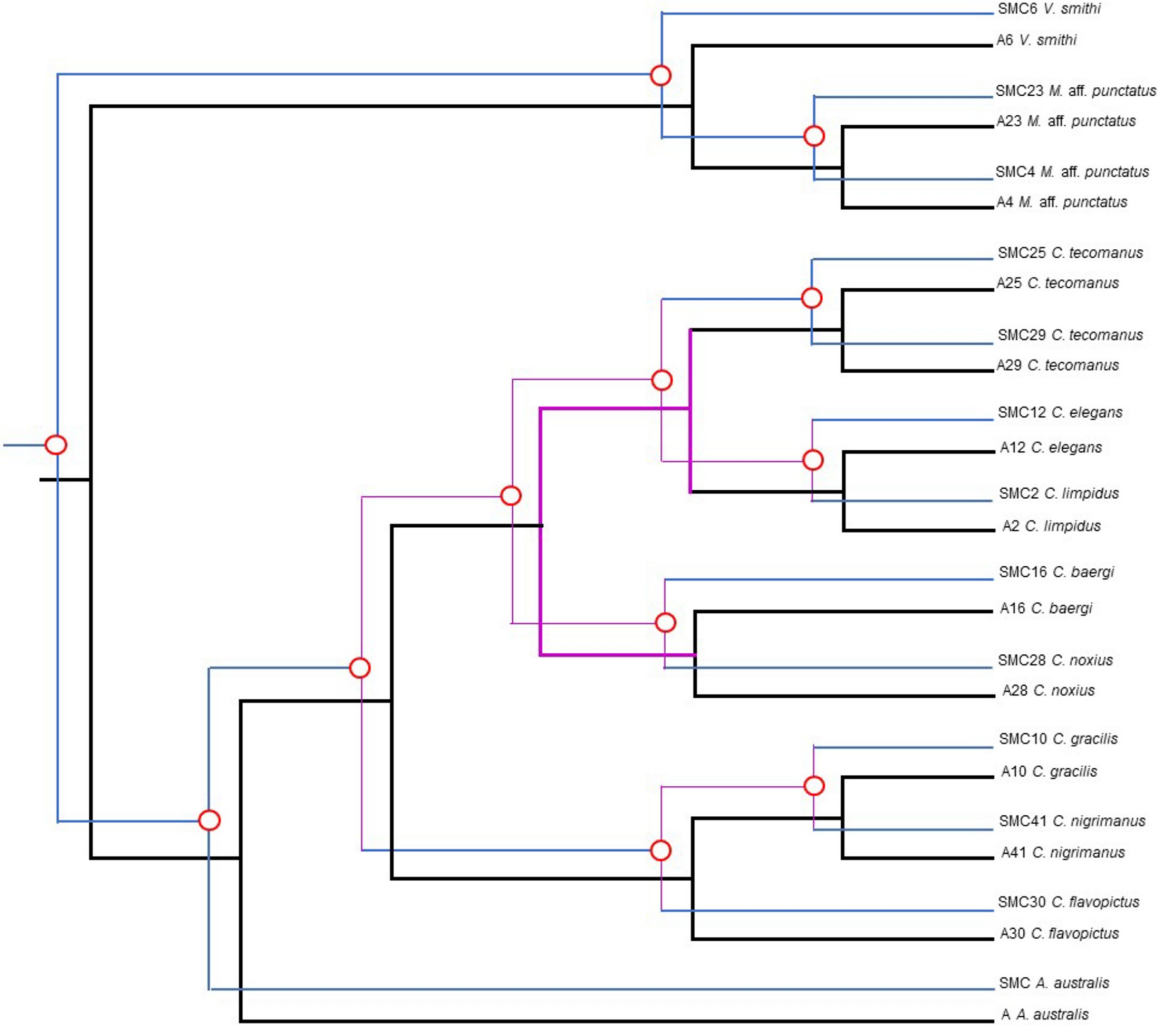
Cophylogenetic reconstruction of scorpion hosts and their SMC symbionts obtained with Jane 4. The individual phylogenies were constructed and inferred as in Fig 3. Black branches delineate the scorpion phylogeny. Blue branches represent SMC phylogeny. The polytomies formed after collapsing nodes with bootstrap values lower than 50% are shown in purple. Red circles outlined at the nodes indicate suggested cospeciation events.

The SMC 16S rRNA phylogeny from Vaejovidae and Buthidae mimics the scorpion phylogeny (Fig 3 and S2 Fig). The phylogenies of scorpion and SG1 showed a similar “mirror” pattern (Fig 3), but the low number of positive species limited a statistical cospeciation analysis, which could be performed with the SMC phylogeny. Some nodes of the SMC 16S rRNA gene phylogeny had very low bootstrap values and very short branches (Fig 3). Therefore, we decided to collapse nodes with 50% or lower bootstrap values, resulting in some polytomies that can be analysed with Jane 4 (Fig 4). This analysis showed several possible cophylogenies, all of which suggested cospeciation in all the nodes (Fig 5). No other evolutionary events such as host-switches, losses, duplications or failure to diverge were revealed by Jane. We performed random reconstructions and compared the total cost values. Of a total of 1000 randomizations, 99.5% had a total cost value higher than that of our predicted results, suggesting that the reconstructions obtained may not be attributed to randomness.

To explore the alternative hypothesis that the pattern of SMC presence and distribution might not be entirely explained by cospeciation, we tested whether the location and geographical distance between collection localities were biasing our results. Linear geographical distances between collection sites did not correlate with the positive SMC amplifications (Mantel rho = 0.04999, p= 0.279). This means that there is no specific geographical region more prone to be inhabited by scorpions with SMC. Linear distances did not show a significant correlation with scorpion genetic distances (Mantel rho = 0.3873, p = 0.006), as we can find different species cohabiting single regions. Additionally, SMC genetic distances did not correlate with linear distances either (Mantel rho = 0.3506, p = 0.021). However, genetic distances between SMC and scorpions do have a significant correlation (Mantel statistic r: 0.9478 significance: 0.001). This evidence suggests that closely genetically related scorpions will host closely related SMC and vice versa, as expected under the cospeciation hypothesis.

DNA from embryos of different scorpion species was also used as a template for PCR amplifications. SG1 was amplified from gut and embryos in one female *M*. aff. *punctatus* (A20). Regarding SMC, we amplified it from gut and embryos in a female *C*. *noxius* (A28) (Table 1). These results indicate a probable transmission of the symbionts to the embryos; however, it remains unclear whether the bacteria are found inside the embryos or over them.

## Discussion

Two scorpion species, *C*. *limpidus* and *V*. *smithi*, were previously found to harbor a *Mycoplasma* novel clade (SMC) and a novel Mollicutes (SG1) restricted to *V*. *smithi* [48]. In this study, a more extensive scorpion sampling approach was used to explore whether the Mollicutes bacteria extend to other scorpion species. A total of 23 scorpion morphospecies belonging to five families were sampled.

SMC was present in some specimens from Vaejovidae and Buthidae families, and SG1 was found only in Vaejovidae (all *Vaejovis* individuals and two out of three *Mesomexovis* species). The Citri-Chrysopicola-Mirum *Spiroplasma* sequences that were found in scorpions (from *Diplocentrus duende* A44 and *Androctonus australis*) were related to common insect symbionts [67] and their high identities suggest recent horizontal acquisition.

SMC lineages in the Mollicutes tree mirrored the scorpion phylogeny which indicated a possible cospeciation processes. Although some external nodes of the Mollicutes tree had low bootstrap support value when SMC sequences were included, scorpion *Mycoplasma* clustering within the clade is stable and highly supported. The phylogenetic tree constructed with the *rpoB* gene shifted the SMC position to the Hominis group. A recent phylogenomic Mollicutes reconstruction using SMC genomic sequences recovered from *Centruroides vitattus* and *Centruroides sculpturatus* placed SMC within the Hominis group [68]. A more accurate phylogenetic position of SMC will be achieved as more sequences become available.

Jane cophylogeny analyses supported the possibility of cospeciation between SMC and scorpions. The suggested cospeciation events indicate that SMC is an ancient symbiont within scorpions. Additionally, Mantel tests showed that the presence of SMC did not correlate with geographic region, but with host genetic distances as expected with cospeciation. The most recent phylogenomic reconstruction of scorpions placed Buthidae in the parvorder Buthida and Vaejovidae in the parvorder Iurida [50]. Based on the chelicerate hemocyanins phylogeny, these parvorders seemingly separated ~120 million years ago [69–71]. If the first bacterial infection of SMC took place in the common ancestor of the two parvorders, the lack of bacterial gene amplifications in other scorpion families beyond Vaejovidae and Buthidae is intriguing and could be explained by primers biased to existing SMC 16S rRNA sequences, low bacterial abundances, or PCR inhibitors. Shotgun metagenomic sequencing of multiple species, including Vaejovidae and Buthidae, as well as an analysis of more specimens of each scorpion species will be needed to clearly determine the presence or absence of Mollicutes in other scorpions. Dissecting scorpions without any starving laboratory treatment could have precluded enrichment of SMC [48,72]. The low number of collected specimens belonging to Carboctonidae and Euscorpiidae led to inconclusive negative results for these groups.

Embryos of two scorpion species, *M*. aff. *punctatus* (A20) and *C*. *noxius* (A28), were positive for SG1 and SMC respectively, indicating that these symbionts are probably transmitted vertically. Vertical transmission would support the cospeciation mechanism, as has been reported for insects and their bacterial endosymbionts [24, 43].

Besides Arthropoda, vertical transmission of associated bacteria has also been observed in other invertebrates as in Porifera, Bivalvia, Ascidiacea, Bryozoa, Oligochaeta, Cephalopoda and Nematoda [1] and cospeciation has been suggested in some of them [73–76]. Some invertebrates may also present a mixed-mode of transmission (from the environment and maternally inherited) [1,77,78].

Pentatomomorpha insects have evolved post-hatch mechanisms to transmit their gut symbionts to the next generation [37]. It is known that mammals may pass their gut symbionts to neonates through breast milk [79] among other strategies; animals may do so also by trophallaxis (direct transfer of food or fluids from one individual to another), or coprophagy [77–83]. We do not know how scorpions may transmit their symbionts to their offspring. A recent study in *Androctonus australis* found that SMC was present in scorpion’s gonads adding evidence of a probable vertical transmission [49]. To further study the transmission of these bacteria, fluorescent *in situ* hybridization should be done.

Mollicutes is a bacterial class widely associated with plants, animals, and fungi [84–86]. Among Mollicutes, mycoplasmas have been primarily recognized as vertebrate pathogens or opportunistic organisms [87,88]. Spiroplasmas are commensals or pathogens in plants and insects [89]. Over the last years, novel Mollicutes lineages related to the *Mycoplasma* and *Spiroplasma* genera have been discovered in different invertebrate animals such as jellyfishes [90], and deep sea [91,92] and terrestrial isopods [93]. Many *Mycoplasma* and *Spiroplasma* species are considered fastidious bacteria due to their complex nutrient requirements [94].

Here we suggest that the SMC and SG1 symbionts are not generalists, since they are specifically found in their host species; we consider that they may be beneficial to scorpions. Although Mollicutes are not generally recognized as mutualist organisms, some examples have been described recently, in which they can provide benefits to their hosts by conferring protection against viruses [92,95] and parasites [96]. Additionally, the proportion of SMC increases in food-deprived scorpions compared to recently captured or laboratory-fed individuals [48], suggesting that these bacteria are not transient food-derived microbiota, but have a stable relationship with scorpions. An evolutionary process of cospeciation is suggested by a host-symbiont mirror phylogeny, as well as more in-depth cophylogenetic analyses. Importantly, these bacterial symbionts were found among a heterogeneous group of scorpion species, differing in geographical locations and ecomorphotypes (*sensu* [97]) along with intrinsic differences in physiologic traits (e.g. metabolic rates) and behavioural characteristics (e,g, feeding or burrowing) [97].

Bacterial symbionts have been recognized as important participants in the physiology, ecology, and evolution of arthropods [43]. Here we showed a broader species distribution of novel Mollicutes lineages in scorpions and a possible cospeciation process.

## Supporting information

S1 Fig Uncollapsed 16S rRNA Mollicutes phylogeny. 16S rRNA phylogeny of Mollicutes showing all sequences used for constructing the collapsed phylogeny in Fig 2.
(TIF)

S2 Fig *rpoB* Mollicutes phylogeney. *rpoB* gene phylogeney of Mollicutes showing all sequences except the scorpion groups SMC and SG1, which are shown collapsed.
(TIF)

S3 Fig Comparison of scorpion and symbiont phylogenies. Phylogenies described in Fig 3 reconstructed with Bayesian Inference. (A) Scorpion phylogeny with concatenated 16S rRNA, CO1 and 28S rRNA genes. (B) SMC 16S rRNA gene phylogeny. (C) SG1 16S rRNA gene phylogeny.
(TIF)

S4 Fig Single marker gene scorpion phylogenies. Phylogenies for each of the three marker genes performed with maximum likelihood. Substitution models used were T92 + G for 16S rRNA, GTR + G + I for CO1, and HKY for 28S rRNA. Colors for each genus or clade are as in Fig 3.
(TIF)

S1 Table Primers used for *rpoB* PCR amplification. (DOCX)

S2 Table False positive sequences obtained by PCR with primers Myco65F and Myco1429R for 16S rRNA of SMC. (DOCX)

S1 Fasta file Fasta file of Mollicutes *rpoB* sequences obtained from *Vaejovis smithi* and *Mesomexovis* aff. *punctatus*. (FASTA)
