# Supplementary figures and images for: Cophylogenetic analysis suggests cospeciation between the Scorpion Mycoplasma Clade symbionts and their hosts
Source: PLoS One. 2019 Jan 9;14(1):e0209588. doi: 10.1371/journal.pone.0209588 (PMC6326461; doi:10.1371/journal.pone.0209588)

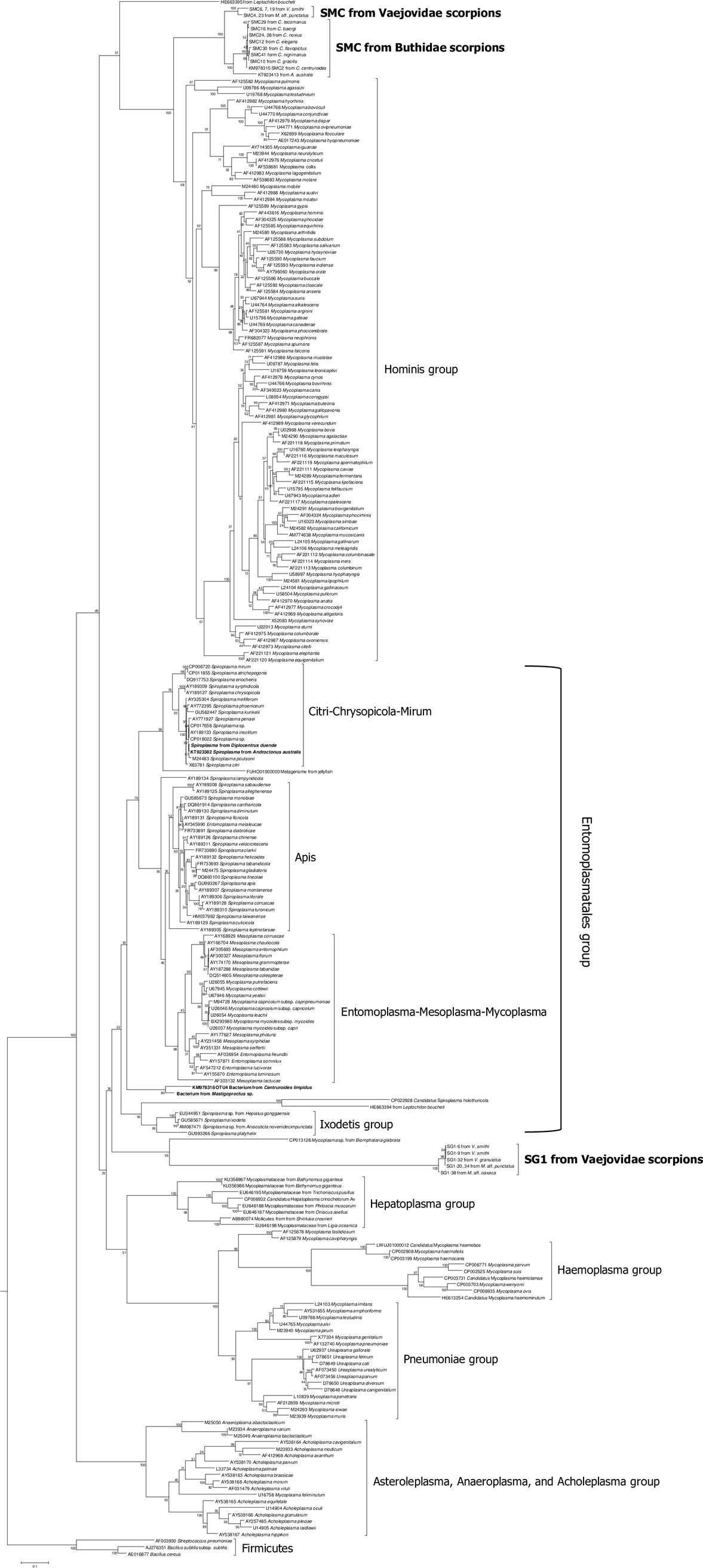

Supplement: S1 Fig — 16S rRNA phylogeny of Mollicutes showing all sequences used for constructing the collapsed phylogeny in Fig 2. (TIF) [file pone.0209588.s001.tif]

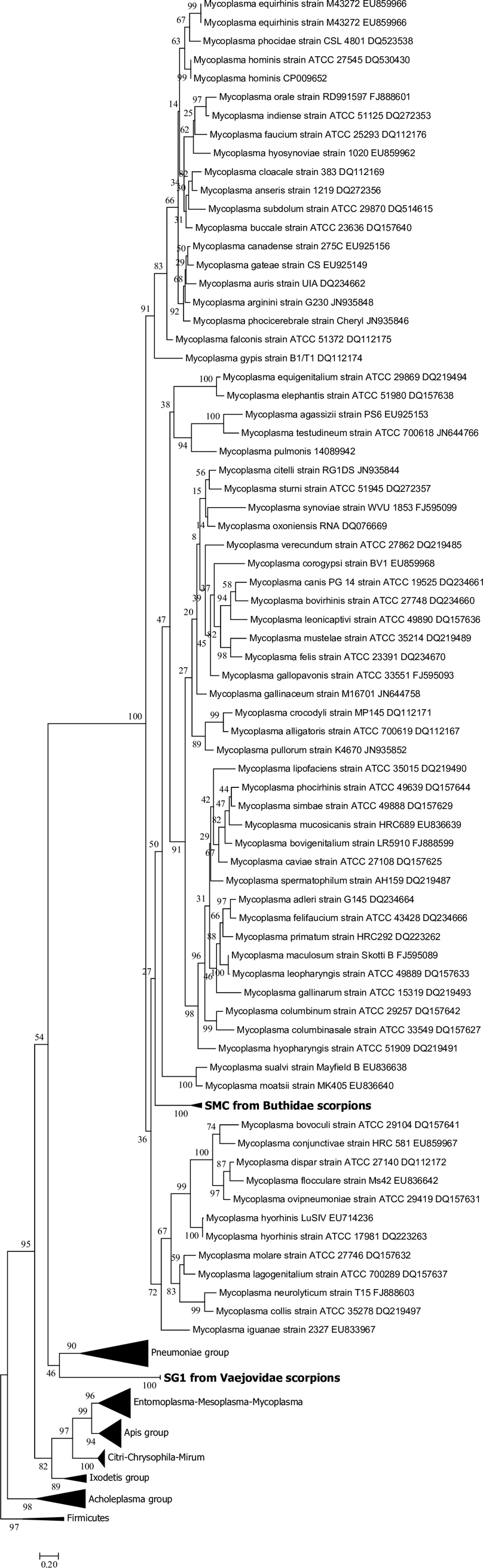

Supplement: S2 Fig — rpoB gene phylogeney of Mollicutes showing all sequences except the scorpion groups SMC and SG1, which are shown collapsed. (TIF) [file pone.0209588.s002.tif]

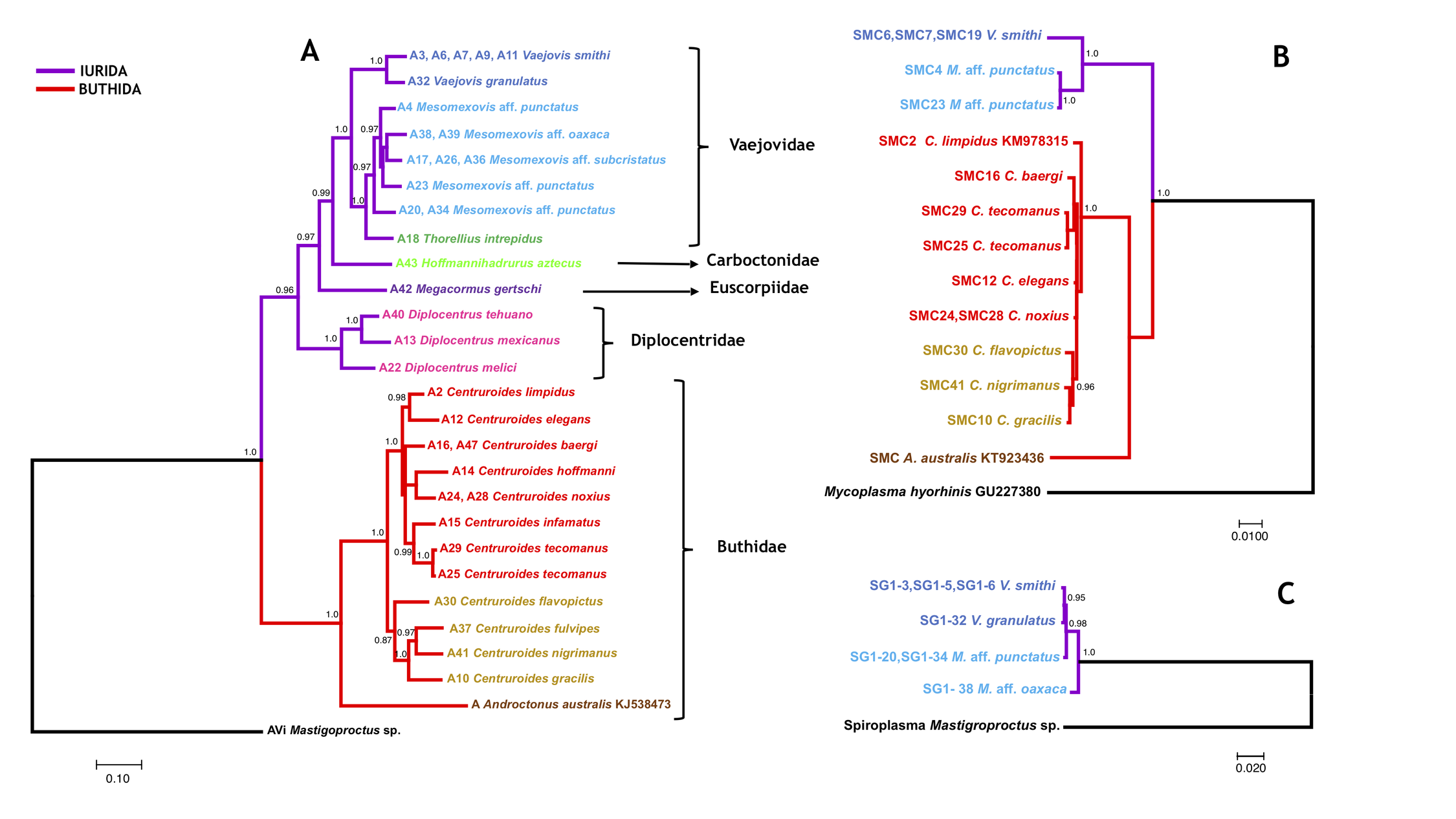

Supplement: S3 Fig — Phylogenies described in Fig 3 reconstructed with Bayesian Inference. (A) Scorpion phylogeny with concatenated 16S rRNA, CO1 and 28S rRNA genes. (B) SMC 16S rRNA gene phylogeny. (C) SG1 16S rRNA gene phylogeny. (TIF) [file pone.0209588.s003.tif]

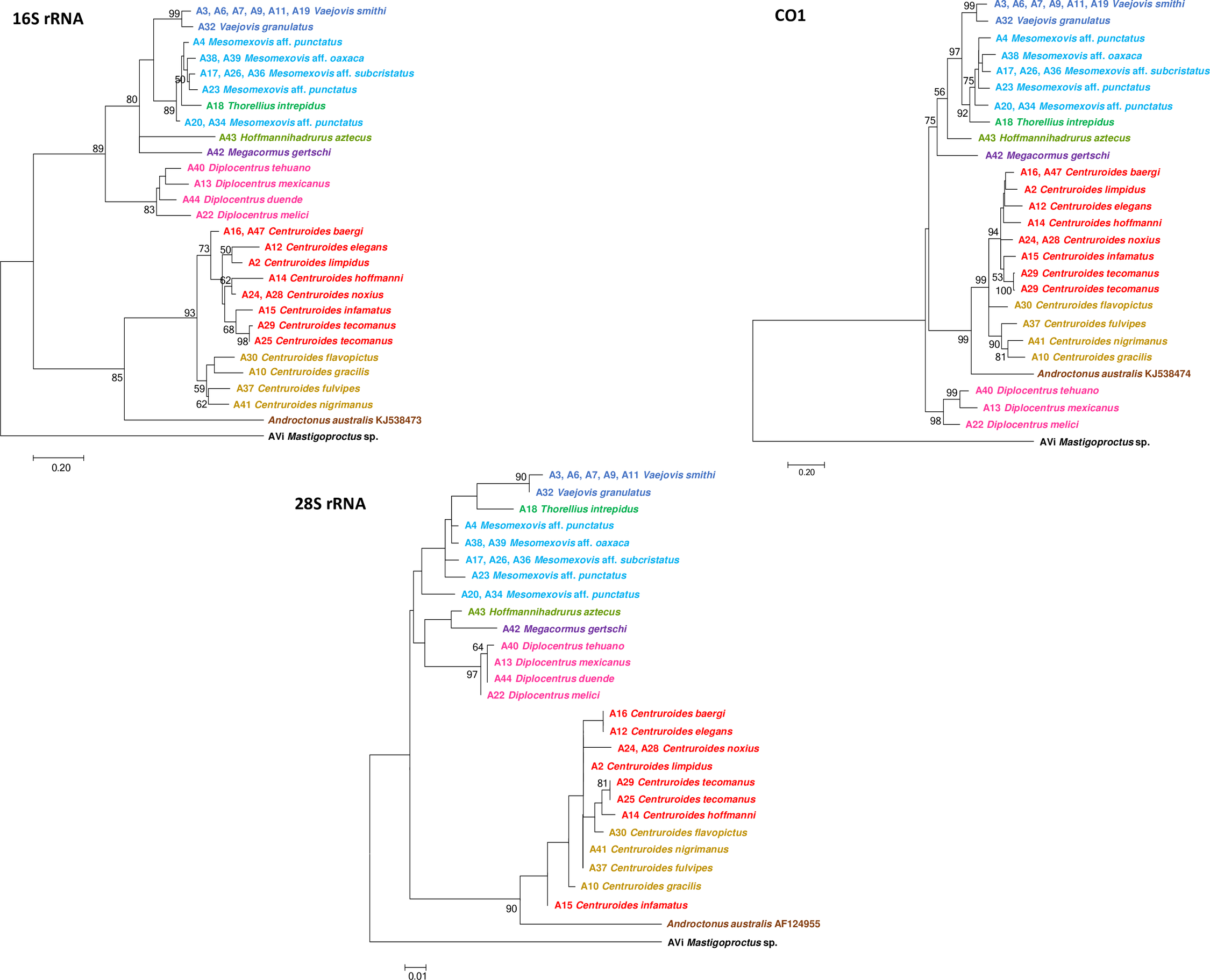

Supplement: S4 Fig — Phylogenies for each of the three marker genes performed with maximum likelihood. Substitution models used were T92 + G for 16S rRNA, GTR + G + I for CO1, and HKY for 28S rRNA. Colors for each genus or clade are as in Fig 3. (TIF) [file pone.0209588.s004.tif]
