# Supplementary material for: Cophylogenetic analysis suggests cospeciation between the Scorpion Mycoplasma Clade symbionts and their hosts
Source: PLoS One. 2019 Jan 9;14(1):e0209588. doi: 10.1371/journal.pone.0209588 (PMC6326461; doi:10.1371/journal.pone.0209588)
Supplement: S1 Table — (DOCX) [file pone.0209588.s005.docx]

**Table S1.** Primers used for *rpoB* PCR amplification.

| **Primer** | **Sequence (5’ – 3’)** | **Gene** | **PCR size (pb)** | **Annealing tempera-ture (^o^C)** | **Extension time (minutes)** | **Reference** |
| --- | --- | --- | --- | --- | --- | --- |
| rpoB228F (P1) | CTTATGGGCGCTAATATGCAA | SMC rpoB | ~890 | 48 | 1 | This study |
| rpoB1113R (P4) | ATTTAAAAACGGCATATCTTC |  |  |  |  |  |
| SG1rpoB419F (BF) | TCCAATTGAATCGCCTGAAGG | SG1 | ~1500 | 43 | 1:45 | This study |
| SG1rpoB2055R (BR) | ACATCTTTGACCACCATTTTG | rpoB |  |  |  |  |
