# Supplementary material for: Cophylogenetic analysis suggests cospeciation between the Scorpion Mycoplasma Clade symbionts and their hosts
Source: PLoS One. 2019 Jan 9;14(1):e0209588. doi: 10.1371/journal.pone.0209588 (PMC6326461; doi:10.1371/journal.pone.0209588)
Supplement: S2 Table — (DOCX) [file pone.0209588.s006.docx]

**Table S2.** False positive sequences obtained by PCR with primers Myco65F and Myco1429R for 16S rRNA of SMC.

| **ID** | **Species** | **16S rRNA sequence with highest score** | **Identity**  **%** |
| --- | --- | --- | --- |
| A13 | *Diplocentrus mexicanus* | *Streptococcus pneumoniae* strain K31 MF578793 | 99 |
| A18 | *Thorellius intrepidus* | *Streptococcus mitis* strain K14 MF578779 | 99 |
| A2 | *Centruroides limpidus* |  |  |
| A14 | *Centruroides hoffmanni* | Uncultured Spirochaetaceae clone Clld2-10 (from *Centruroides limpidus*) KM978314 | 100 |
| A15 | *Centruroides infamatus* | *Dysgonomonas oryzarvi* strain Dy73 NR_113063 | 100 |
